# Design and rationale of the IN CONTROL trial: the effects of real-time continuous glucose monitoring on glycemia and quality of life in patients with type 1 diabetes mellitus and impaired awareness of hypoglycemia

**DOI:** 10.1186/s12902-015-0040-3

**Published:** 2015-08-21

**Authors:** Cornelis A.J. van Beers, Susanne J. Kleijer, Erik H. Serné, Petronella H. Geelhoed-Duijvestijn, Frank J. Snoek, Mark H.H. Kramer, Michaela Diamant

**Affiliations:** Diabetes Center, Department of Internal Medicine, VU University Medical Center, Amsterdam, HV 1081 The Netherlands; Department of Internal Medicine, Medical Center Haaglanden, The Hague, The Netherlands; Department of Medical Psychology, VU University Medical Center, Amsterdam, The Netherlands; Department of Medical Psychology, Academic Medical Center, Amsterdam, The Netherlands

## Abstract

**Background:**

Hypoglycemia is the main side effect of intensified insulin therapy in type 1 diabetes and recognized as a limitation in achieving glycemic targets. Patients with impaired awareness of hypoglycemia have a threefold to sixfold increased risk of severe hypoglycemia. Real-time continuous glucose monitoring may help patients with type 1 diabetes to achieve better glycemic control with less hypoglycemic episodes. Accordingly, one may hypothesize that particularly type 1 diabetes mellitus patients with impaired awareness of hypoglycemia will profit most from this technology with improvements in their quality of life. However, this has not yet been established. This trial aims to study the effect of real-time continuous glucose monitoring on glycemia and quality of life specifically in type 1 diabetes mellitus patients with established impaired awareness of hypoglycemia.

**Methods/design:**

This is a two-center, randomized, cross-over trial with a 12-week wash-out period in between intervention periods. A total of 52 type 1 diabetes mellitus patients with impaired awareness of hypoglycemia according to Gold et al. criteria will be randomized to receive real-time continuous glucose monitoring or blinded continuous glucose monitoring for 16 weeks. After a wash-out period, patients will cross over to the other intervention. The primary outcome measure is time spent in euglycemia. Secondary outcomes include (diabetes-specific) markers of quality of life and other glycemic variables.

**Discussion:**

It remains unclear whether patients with type 1 diabetes and impaired awareness of hypoglycemia benefit from real-time continuous glucose monitoring in real-life. This study will provide insight into the potential benefits of real-time continuous glucose monitoring in this patient population.

**Trial registration:**

Clinicaltrials.gov: NCT01787903.

**Electronic supplementary material:**

The online version of this article (doi:10.1186/s12902-015-0040-3) contains supplementary material, which is available to authorized users.

## Background

Type 1 diabetes mellitus (T1DM) constitutes about 10-15 % of total diabetes rates and its incidence has been increasing worldwide at an alarming rate of 3-5 % per year [1]. T1DM is associated with an increased risk of microvascular and macrovascular co-morbidities. The Diabetes Control and Complication Trial/Epidemiology of Diabetes Interventions and Complications (DCCT/EDIC) studies have shown that intensive versus conventional glycemic control results in a reduction of microvascular [2] but also macrovascular complications [3]. However, the DCCT also demonstrated that intensive treatment associates with a considerably increased rate of hypoglycemia [2]. With incidences of approximately 2 per patient per week [4–6] for mild (i.e. self-treated) hypoglycemia, and 0.1-1.5 per patient year [2, 5–9] for severe hypoglycemia (SH), hypoglycemia is both the main limitation in achieving glycemic targets and the main side effect of intensified insulin therapy in T1DM [10, 11]. The American Diabetes Association (ADA) defines SH as an event requiring assistance of a third party, with symptoms of neuroglycopenia and recovery of neurological symptoms after restoration of plasma glucose [12].

Hypoglycemia is both a physical and psychological burden [13, 14]. It can interfere with every aspect of daily life, such as sleep, exercise, driving or otherwise travelling, but also social interactions and even employment [13]. Patients worry about having episodes of SH or getting the late complications of T1DM [6], and there is always the possibility of a hypoglycemic episode leading to a coma [6]. Recurrent hypoglycemia may promote the development of impaired awareness of hypoglycemia (IAH) by decreasing the glycemic threshold in the brain required for the activation of the autonomic system [15]. Consequently, by the time the glucose level is low enough to elicit symptoms, patients fail to recognize them due to neuroglycopenia. To date, no consensus on a satisfactory definition of IAH exists. We defined IAH as the diminished ability to recognize the onset of hypoglycemia [14]. Hypoglycemia awareness can be assessed by using self-rating questionnaires. The Gold method consists of one question: “do you know when hypoglycemia is commencing?”. The state of hypoglycemia awareness is then assessed by using a 7-point visual analogue scale, with 1 representing “always aware” and 7 representing “never aware”. A score of ≥4 suggests impaired awareness of hypoglycemia [16]. The Clarke method consists of eight questions identifying the respondents exposure to episodes of (mild and severe) hypoglycemia and examining the glycemic threshold for hypoglycemia. Impaired awareness of hypoglycemia is implied if the respondent has a score of ≥4 [17]. Both scoring systems show good performance in adults [18]. Impaired awareness of hypoglycemia renders patients at a threefold to sixfold increased risk of SH which considerably hampers their quality of life [13, 16, 19]. In addition, hypoglycemia can be fatal, with hypoglycemia mortality estimates ranging from 4 to 10 percent of deaths of patients with type 1 diabetes [20, 21]. Furthermore, a recent observational study showed that T1DM patients with an glycated hemoglobin level of 6.9 % or lower had a twice increased risk of death from any cause and cardiovascular causes compared with matched controls [22].

In 2006, real-time continuous glucose monitoring (RT-CGM) was introduced to assist patients in their self-management of blood glucose [23]. Real-time continuous glucose monitoring systems measure interstitial glucose levels and provide this information every five or ten minutes, with a delay of approximately 8 to 15 minutes [24–26]. The added value lies in the display of trends and alarms that can be set to warn for impending hypo- or hyperglycemia [27, 28]. RT-CGM is associated with an improvement of glycemic control [27, 29–41] and shorter duration of hypoglycemic episodes [42–44]. However, the effect of conventional RT-CGM (i.e. without automated insulin suspension) specifically in patients with IAH has not yet been established in a real life setting [45]. In most studies either recent severe hypoglycemia was among the exclusion criteria [29] or the frequency of severe hypoglycemia at baseline was not mentioned [37, 40, 46]. Studies done in subjects with normal hypoglycemia awareness already indicate that use of RT-CGM might improve the impact of T1DM on daily life [28] and might increase treatment satisfaction [47], although an effect on the fear of hypoglycemia is not apparent [48]. Since experiencing the issues mentioned above might especially be the case for patients with IAH, it is not unlikely that use of RT-CGM might improve the quality of life in those patients.

This trial aims to study a wide range of effects of RT-CGM specifically in T1DM patients with established IAH, regardless of baseline HbA1c. We hypothesize that the use of RT-CGM, relative to a control intervention using masked CGM, will result in improvement of time spent in euglycemia and quality of life in T1DM patients with IAH.

## Methods/design

### Design

**Fig. 1:**
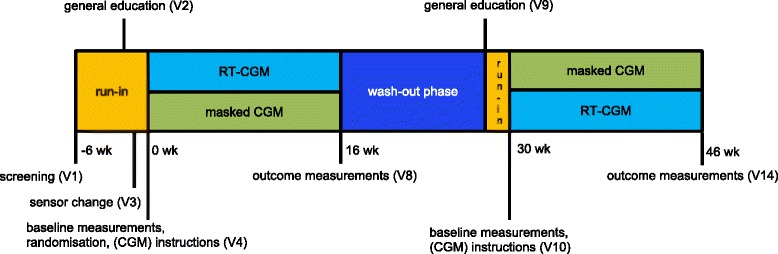
General study plan

This is a two-center, randomized, cross-over trial with a 12-week wash-out period in between intervention periods (Fig. 1). Ethical approval has been granted by the medical ethical committee of the VU University Medical Center (VUMC).

### Recruitment

Subjects will be recruited from the outpatient clinic of the VUMC, Amsterdam and the Medical Center Haaglanden (MCH), The Hague, The Netherlands, from outpatient clinics at affiliated hospitals, but also from regional hospitals and via advertisements in local newspapers. Responders will be sent written extensive patient information. Written informed consent will be obtained from the subjects prior to any trial related procedure.

### Study population

Fifty-two subjects with T1DM and IAH will be enrolled. Because patients with IAH often are already in good to moderate control in terms of HbA1c, patients will be included regardless of their HbA1c.

#### Inclusion criteria

- T1DM, diagnosed according to ADA criteria [49] regardless duration.
- Use of multiple daily injections of insulin or continuous subcutaneous insulin infusion. (Participants may not change their treatment method during the study.)
- Age between 18 and 75 years (inclusive).
- IAH according to the questionnaire by Gold et al. [16].
- Performing self-monitored blood glucose measurements (SMBG) at least 3/day or 21/week.

#### Exclusion criteria

- History of (recent) major renal, liver, or (ischemic) heart disease (including cardiac conduction disorders).
- Current untreated proliferative diabetic retinopathy.
- Current (treatment for) malignancy.
- Current use of non-selective beta-blockers.
- Current psychiatric disorders, including schizophrenia, bipolar disorder, anorexia nervosa or bulimia nervosa.
- Substance abuse or alcohol abuse (men >21 units/week, women >14 units/week).
- Current pregnancy or intention to conceive.
- Current use of RT-CGM other than for short term (i.e. diagnostic use or use shorter than 3 consecutive months).
- Hearing or vision impairment hindering perceiving of glucose display and alarms, or otherwise incapable of using a (RT-)CGM, in the opinion of the investigator.
- Poor commandment of the Dutch language or any (mental) disorder that precludes full understanding of the purpose and instructions of the study.
- Participation in another clinical study.
- Known or suspected allergy to trial product or related products.

### Study plan

A detailed overview of study visits, telephone consultations and procedures is given in Additional file 1. At each visit, a recent history will be taken, including e.g. complaints, adverse events, changes in medication treatment regime and the occurrence of SH (i.e. requiring assistance of a third party). Weight and vital signs will be measured at each visit. Outcome measurements will be performed at baseline and after each 16-week intervention phase (Fig. 1).

### Screening

Hypoglycemia awareness will be assessed using the questionnaire developed by Gold et al. and by Clarke et al. [16, 17]. The frequency and severity of severe hypoglycemia will be assessed using the self-report questionnaire according to Langan et al. [50]. Subsequently, a complete medical and socio-economic history will be taken and a complete physical examination will be performed. Participants will be examined for the presence of diabetes-related (microvascular) complications. Thus, plasma creatinine, calculated estimated glomerular filtration rate (eGFR) and albumin-creatinine ratio (ACR) in a random urine sample will be measured to evaluate diabetic nephropathy [51, 52]. The presence/severity of diabetic sensorimotor polyneuropathy will be quantified by the Modified Toronto Clinical Scoring System [53]. For the assessment of vibration sense, the vibration perception threshold will be quantified using a neurothesiometer [54]. The presence/severity of diabetic retinopathy (DRP) will be assessed based on the relevant medical history and a recent (<6 months) evaluation by the patient’s ophthalmologist. In the absence of this information, patients will be either requested to visit their ophthalmologist prior to study onset or they will be referred for fundus photography [55]. Blood will be collected to ascertain in- and exclusion criteria and for baseline measurement.

### Run-in phase

A 5-week run-in phase, starting after inclusion, will be used to allow for study effects and enable diabetes- and study-related education. All participants will receive approximately 30 minutes of individual education in the principles of diabetes management, including basic principles of standardized SMBG, glucose fluctuations, insulin and carbohydrates, hyper- and hypoglycemia, impaired awareness of hypoglycemia and RT-CGM. In case a participant does not use the technique of carbohydrate counting, no education on this subject will be given in order to prevent confounding. Participants will be equipped with a masked CGM device to be worn for two weeks to gather baseline data and to familiarize them with longer-term wearing of this type of device. During follow-up visits, there will be an ongoing evaluation of diabetes self-management and knowledge of diabetes management, as this is part of standard diabetes care. A test of knowledge will not be used.

### Intervention phase

#### Randomization

After successful participation in the run-in phase, participants will be randomized, using block randomization (allocation 1:1), to the first intervention period. A sealed envelope for each participant will be drawn and opened by qualified study staff. In addition, the block size will be determined by the institutional trial pharmacist. Study staff and participants will not be blinded for treatment order, as this is an open intervention study. When allocated to RT-CGM, participants will receive additional education on how to use RT-CGM and the accompanying specialized software. Participants will be asked to upload their RT-CGM-data before each follow-up visit. When allocated to masked CGM, participants will continue to wear their CGM device from the run-in phase. Participants are encouraged to wear the assigned study devices continuously.

#### Follow-up visits and telephone consultations

During the intervention periods there will be monthly follow-up visits and telephone consultations involving inquiry after e.g. actual complaints/symptoms, episodes of (severe) hypoglycemia, study device use and related technical issues, and medication using a checklist. During both intervention periods, research physicians will guide participants in concordance with the ADA Standards of Medical Care in Diabetes [56]. The guidance will be equal during both intervention periods. During the visits, therapy adjustments will be discussed and logged, based on RT-CGM-data in the RT-CGM group or SMBG-data in the masked CGM group. No treatment or insulin titration protocol will be used, neither will SMBG use be standardized, in order to avoid additional interventions.

#### Wash-out phase

After the first intervention phase, participants will enter a 12-week wash-out phase, during which they will only receive two-weekly telephone consultations for taking recent histories and monitoring of potential adverse events. At the end of the wash-out period, general diabetes and CGM education will be given similar to the first run-in phase. Also, participants will start to wear masked CGM again for two weeks to gather baseline data for the second intervention period.

### Endpoints

#### Primary endpoint

The mean difference in time spent in euglycemia (interstitial glucose range >3.9 mmol/L - ≤10.0 mmol/L), expressed as hours/day between the two intervention periods. The specific ranges of euglycemia are based on the definition of the ADA and comparing literature [12, 44].

#### Secondary endpoints

- (Diabetes-specific) markers of quality of life, as assessed with questionnaires covering: diabetes-related emotional distress (PAID-5 (Cronbach’s α = 0.86) [57]), fear of hypoglycemia (HFS-2 (Cronbach’s α = 0.94) [58, 59]), diabetes self-efficacy (CIDS (Cronbach’s α = 0.94) [60]), generic health-status (EQ5D (Cronbach’s α = 0.73) [61, 62]) and emotional well-being (Cronbach’s α = 0.82) (WHO-5 [63–65]).
- Other glycemic variables, including HbA1c, time spent in hypo- and hyperglycemia ranges, frequency of severe (i.e. requiring assistance of a third party) and mild (i.e. self-treated) hypoglycemia (assessed by analyzing (RT-)CGM data) and duration of hypoglycemia.
- Changes in hypoglycemia awareness score according to Gold et al. [16].
- Glycemic variability. Glucose variability (defined as inter-day and intraday glycemic variability), will be calculated as Mean Of Daily Differences and Continuous Overall Net Glycemic Action, using all (RT-)CGM values during the 16 week intervention period [66, 67].

### Exploratory endpoints

- Autonomic nervous system function. The functioning of the autonomic nervous system will be indirectly assessed by analysing heart rate variability and blood pressure changes during four of the five Ewing’s standardized cardiovascular reflex tests: the Valsalva ratio, the 30:15 ratio on standing up, the maximum-minimum heart rate during deep breathing and postural blood pressure change [68–71]. The heart-rate variability will be assessed using non-invasive, automated beat-to-beat blood pressure and ECG recordings (Nexfin®, BM Eye, Amsterdam, the Netherlands). that will be analyzed by dedicated software, automatically calculating HRV indices for both the time-domain and frequency-domain variables. Quality control will be performed by research staff by visual inspection of the data. Data will be excluded from analysis if >5 % of the measured beats are extrasystoles. No agreement exists on the number of abnormal cardiovascular tests required to reach the diagnosis of cardiovascular autonomic neuropathy [71]. However, an abnormality of more than one test, on more than one occasion, is indicative of (cardiovascular) autonomic dysfunction [71, 72].
- Duration of sensor wear.
- Changes in hypoglycemia awareness score according to Clarke et al. [17].
- Satisfaction with use of RT-CGM, assessed by the CGM-SAT questionnaire [73].

### Study devices

The MiniMed Paradigm® Veo™ System (Medtronic, Northridge, CA) will be used as RT-CGM device. The system consists of a continuous Enlite™ glucose sensor, a MiniLink^™^ transmitter and the Paradigm® Veo™ System [74, 75]. The sensor is a membrane-covered enzyme coated electrode placed through the skin into the subcutaneous space using an auto-insertion device. This sensor has to be changed every 6 days . The wireless MiniLink^™^transmitter is a small rechargeable device that is connected to the glucose sensor and sends glucose data wirelessly to the monitor every 5 minutes, 24 hours a day. The monitor shows real-time glucose measurements and provides information regarding changes in glucose levels (trends). Calibration is required every 12 hours. The system allows to set alarms, i.e. alarms for low and high glucose limits, as agreed with the health-care provider, at which the system should alert the patient whenever glucose values are approximating or exceeding these pre-set values. The low-glucose limit during this trial will be pre-set at 4,5 mmol/L and cannot be lowered in order to prevent hypoglycemia. Also, accuracy of glucose sensors decreases in hypoglycemic ranges [76, 77]. Low-glucose suspension function will not be used. Finally, the system shows participants their real-time glucose values and patterns, and allows for adjustment of treatment and/or lifestyle accordingly.

The masked CGM device that will be used is the iPro™2 Continuous Glucose Monitor (Medtronic, Northridge, CA), which also uses the Enlite™ glucose sensor [74, 75]. Masked CGM- and SMBG-data must be uploaded every week for the purpose of (retrospective) calibration of the iPro™2. Participants will be blinded to the CGM-data. The investigators will review the CGM-data for quality based on duration of sensor wear, number of sensor values, accuracy (mean absolute difference), number of valid calibrations. In case of missing data due to low quality CMG-data, the intervention phase will be extended.

### Statistical considerations

#### Analysis

Intention to treat analysis will include all randomized subjects, including drop-outs. Per-protocol analysis will include only subjects who have completed at least the first four weeks of the first intervention period. The total evaluable sample will consist of all intention-to-treat subjects who complete both intervention periods according to the protocol. Evaluation of (RT-)CGM data will not be performed in an assessor-blinded way. Quality control of the (RT-)CGM data and data analysis will be performed by the same study staff.

Unless otherwise stated, all statistical tests will be conducted at a 2-tailed significance level of 0.05. According to their distribution, the various parameters will be expressed as mean (± SD), median (interquartile range) or number (%). Residual effects will be ascertained before further analysis will be performed using non-parametric tests [78, 79]. The primary endpoint, i.e. mean difference in time spent in the euglycemic range, expressed as hours/day, during the RT-CGM versus the masked CGM phases, will be analyzed per month using mixed model analysis, in order to show a possible effect over time. In the mixed model analysis, time spent in euglycemia will be used as dependent variable. Independent variables will include: treatment arm, interaction between RT-CGM and CGM, and time in months since start of the intervention. Baseline CGM data, gathered during the run-in phase, will be implemented as covariate. In case of carry-over effects and an unequal distribution of treatment method (MDI / CSII) over the intervention orders (RT-CGM – CGM / CGM – RT-CGM), treatment method will also be included as covariate. Post hoc tests will be corrected using Bonferroni correction. If additional testing will reveal potential confounders, adjustments for these factors may necessitate analysis of covariance (ANCOVA). By using mixed model analysis, unequal use of the devices will not necessitate correction, as long as missing data can be considered to be at random.

### Carry-over

To date, little is known about the possible carry-over effects of RT-CGM. Yet such an effect would seem logical considering the expected educational effect of RT-CGM in e.g. the ability of participants to recognize patterns in otherwise undetected hypo- or hyperglycemic events and adjust insulin therapy accordingly. To minimize carry-over effects, a washout period of 12 weeks will be implanted. We consider a 12-week period sufficient for the behavioral modification component to wear off. Additionally, a 12-week time period allows realistic HbA1c changes resulting from patients self-management during that same period to establish a reliable baseline for the second intervention period.

### Sample size calculation

Since current RT-CGM technology may show relatively poor performance in the hypoglycemic range [80], we chose mean time (hours/day) spent in euglycemia, i.e. glucose values ranging >3.9 - ≤10.0 mmol/L, during the study period as our primary end-point. A previously published intervention study using RT-CGM demonstrated a difference of 1.5 hours in time spent in euglycemia between the RT-CGM and control group [44]. In order to detect a difference of 1.5 hours (6.25 % from 24 hours) in time spent in euglycemia, assuming a standard deviation of 3.5, alpha-level of 0.05, power of 80 %, a drop-out rate of 15 % and a correlation of 0.5 between repeated measures (which is due to the cross-over design of the study in which subjects serve as their own control), a sample size of 52 patients will be needed.

## Discussion

Although it seems obvious that T1DM patients with IAH could potentially benefit from RT-CGM, the effect of conventional RT-CGM (without automated insulin suspension) has not yet been established in an ambulatory setting. A hyperinsulinemic hypoglycemic clamp study of Ly et al. showed that 4 weeks of RT-CGM improved epinephrine responses in young T1DM patients with IAH, suggesting that IAH can be restored in adolescents by using RT-CGM [81]. Furthermore, the first observational study performed in patients with IAH demonstrated a clear reduction of SH with RT-CGM use [82], addressing the need for further intervention studies in patients with IAH. A randomized controlled trial of Little et al. demonstrated that IAH can be restored and SH prevented using existing technology [45]. In contrast to expectations, RT-CGM was not superior over self-monitoring of blood glucose by finger prick. However, during this trial, strict insulin titration protocols where used, which may not reflect real-life diabetes management. Although Ly et al. showed that sensor-augmented insulin pump therapy with automated insulin suspension reduced the frequency of SH significantly in T1DM patients with IAH [83], this reduction lost significance when 2 outliers, whose rates of hypoglycemia were higher at baseline, were removed from analysis. Hence, it remains unclear whether T1DM patients with IAH benefit from conventional RT-CGM in real-life.

The study protocol poses some limitations. Hypoglycemia awareness will be assessed using the subjective self-rating questionnaire of Gold et al. [16]. Objective assessments of hypoglycemia awareness, i.e. by using CGM data or by inducing hypoglycemia in a laboratory setting, will not be used. Self-reported hypoglycemia awareness has shown to be reliable in previous studies [16–18, 84] and reflects clinical practice. Also, continuous use of RT-CGM is encouraged, but not mandatory, which could influence the effect of RT-CGM on glycemia [40]. Furthermore, the low alarm value used in this trial is pre-set at 4,5 mmol/L in order to prevent hypoglycemia. In clinical practice however, alarm values should be evaluated and set individually. Moreover, the decrease in sensor accuracy in hypoglycemic ranges could compromise the analysis of glycemia [76, 77]. Finally, participants already using an insulin pump are offered a second device, which could diminish compliance with regards to logging SMBG values, meals, insulin and activities. This could affect the value of interpreting the RT-CGM-data retrospectively.

With this study, we expect to gain a better understanding of the potential benefits of RT-CGM technology in a sample of patients with T1DM complicated by impaired hypoglycemia awareness. Also, the study is likely to yield clinically relevant information to help further improve targeted intervention strategies to help these patients improve their diabetes self-management and quality of life.

## Additional file

Additional file 1:
**Table S1.** Overview of visits, telephone consultations and outcome assessments. (XLS 34 kb)

## Abbreviations

ADA: American Diabetes Association
ACR: Albumin-creatinine ratio
CGM: Continuous glucose monitoring
CGM-SAT: Questionnaire for satisfaction with use of CGM
CIDS: Confidence in diabetes self-management
DCCT/EDIC: The diabetes control and complication trial/epidemiology of diabetes interventions and complications
DRP: Diabetic retinopathy
EQ-5D: EuroQol-5D
eGFR: estimated glomerular filtration rate
HbA1c: Glycosylated hemoglobin
HFS: Hypoglycemia fear scale
IAH: Impaired awareness of hypoglycemia
MCH: Medical Center Haaglanden
PAID-5: Problem areas in diabetes scale 5-items
QoL: Quality of life
RT-CGM: Real-time continuous glucose monitoring
SH: Severe hypoglycemia
SMBG: Self-monitoring of blood glucose
T1DM: Type 1 diabetes mellitus
VUMC: VU University Medical Center
WHO-5: World Health Organization-Five Well-Being Index
